# Rapid antiretroviral therapy initiation in low- and middle-income countries: A resource-based approach

**DOI:** 10.1371/journal.pmed.1002723

**Published:** 2019-01-15

**Authors:** Mark W. Tenforde, A. Sarah Walker, Diana M. Gibb, Yukari C. Manabe

**Affiliations:** 1 Division of Allergy and Infectious Diseases, Department of Medicine, University of Washington School of Medicine, Seattle, Washington, United States of America; 2 Department of Epidemiology, University of Washington School of Public Health, Seattle, Washington, United States of America; 3 Botswana-UPenn Partnership, Gaborone, Botswana; 4 Medical Research Council Clinical Trials Unit at University College London, London, United Kingdom; 5 Division of Infectious Diseases, Department of Medicine, Johns Hopkins University School of Medicine, Baltimore, Maryland, United States of America; 6 Infectious Diseases Institute, Makerere University College of Health Sciences, Kampala, Uganda

## Abstract

In an Essay, Mark Tenforde and colleagues advocate continued provision of baseline CD4 cell count testing in HIV care in low- and middle-income countries.

## Major progress has been made, but late entry to care remains common in the HIV “treat all” era

Antiretroviral therapy (ART) has substantially decreased HIV morbidity and mortality in high-income as well as low- and middle-income countries (LMICs). Several randomized trials have demonstrated benefits from starting ART regardless of CD4 count (Table 1) [1–3]; the World Health Organization (WHO) adopted a “treat all” strategy in 2015. Significant attention has been focused on rapidly initiating ART, reflected in the 2017 WHO guidelines, which recommend that ART be initiated within 7 days of HIV diagnosis and on the same day whenever possible [4–6]. Although considerable progress has been made, a significant proportion of patients starting ART in LMICs continue to present with severe immunosuppression, with recent laboratory-based surveillance showing that one-third of South African patients still enter care with advanced HIV infection (CD4 < 200 cells/μL) [7,8]. These late presenters have the highest risk for death, unmasking of opportunistic infections (OIs), and immune reconstitution inflammatory syndrome. The guidelines highlight these patients and state that “people with advanced HIV disease should be given priority for clinical assessment and treatment initiation” [6]. Paradoxically, difficulties in implementing guidance on screening for OIs may result in the greatest delays in ART initiation in this population who are at the most risk.

**Table 1:** Influential clinical studies supporting recommendations.

Study Name	Author (year)	Setting	Findings
Benefit of rapid ART initiation			
HPTN 052	Cohen (2011) [1]	1,763 HIV serodiscordant couples in 9 countries (CD4 350–550 cells/μL) randomized to immediate or delayed ART (≤250 cells/μL or AIDS-related illness)	41% (95% CI 12%–60%) reduction in HIV-related clinical events in partner with HIV in immediate ART arm; 96% (95% CI 73%–99%) reduction in HIV transmission to HIV seronegative partner
START	INSIGHT START (2015) [3]	4,685 ART-naïve adults with HIV in 35 countries (CD4 ≥ 500 cells/μL) randomized to initiate ART at CD4 > 500 cells/μL or < 350 cells/μL (or AIDS-related illness)	57% (95% CI 38%–70%) reduction in serious HIV-related or serious HIV-unrelated events or death in early ART arm; significant reductions in TB, Kaposi’s sarcoma, and malignant lymphoma with early ART
TEMPRANO	TEMPRANO ANRS 12136 (2015) [2]	2,056 ART-naïve adults with HIV in Ivory Coast (CD4 < 800 cells/μL) in 2 × 2 factorial design randomized to (1) immediate ART and 6 months IPT, (2) immediate ART without IPT, (3) delayed ART (until current WHO initiation criteria met) and 6 months IPT, and (4) delayed ART without IPT	44% (95% CI 24%–59%) reduction in primary endpoint (death from any cause, AIDS-defining disease, non-AIDS-defining cancer, or invasive bacterial disease) in early versus delayed ART groups
Benefit of IPT if TB screening negative			
TEMPRANO	TEMPRANO ANRS 12136 (2015) [2]	2,056 ART-naïve adults with HIV in Ivory Coast (CD4 < 800 cells/μL) in 2 × 2 factorial design randomized to (1) immediate ART and 6 months IPT, (2) immediate ART without IPT, (3) delayed ART (until current WHO initiation criteria met) and 6 months IPT, and (4) delayed ART without IPT	35% (95% CI 12%–52%) reduction in primary endpoint (death from any cause, AIDS-defining disease, non-AIDS-defining cancer, or invasive bacterial disease) in IPT versus no-IPT groups including 53% (95% CI 3%–77%) reduction in incident TB
REMEMBER	Hosseinipour (2016) [9]	850 ART-naïve participants with HIV ≥13 years old, with CD4 < 50 cells/μL, and without clinical evidence of TB (suspected or confirmed) from 10 high-TB-burden countries randomized to empiric ATT and ART or IPT and ART	Empiric ATT did not reduce 24-week all-cause mortality compared to IPT alone; IPT and ART were safe when given concurrently in patients with advanced HIV; low (5%) all-cause mortality compared to other randomized controlled trials and observational studies
REALITY	Hakim (2017) [10]	1,805 ART-naïve participants with HIV ≥5 years old, with CD4 < 100 cells/μL starting ART randomized to enhanced prophylaxis (12 weeks IPT, 12 weeks fluconazole, 5 days azithromycin, single-dose albendazole, and continuous cotrimoxazole [trimethoprim-sulfamethoxazole]) versus continuous cotrimoxazole alone; beyond 12 weeks, about half of patients in both arms prescribed IPT	27% (95% CI 2%–45%) reduction in all-cause 24-week mortality in enhanced prophylaxis group compared to standard prophylaxis group; 33% (95% CI 7%–51%) reduction in incident TB up to 48 weeks in enhanced prophylaxis group
Benefit of cotrimoxazole prophylaxis*			
CDC-Uganda cohort	Mermin (2004) [11]	Pre-ART era cohort including 509 Ugandans with HIV of any age that started cotrimoxazole after 5 months and followed for additional 18 months	46% (95% CI 16%–65%) reduction in all-cause mortality with cotrimoxazole compared to period before initiation; 72% (95% CI 60%–81%) reduction in malaria, 35% (95% CI 19%–47%) reduction in diarrheal illness, and 31% (95% CI 2%–52%) reduction in hospital admissions
DART trial cohort	Walker (2010) [12]	Cohort of 3,179 ART-naïve adults with HIV in Uganda and Zimbabwe started on ART in DART trial	59% (95% CI 35%–73%) reduction in mortality in participants taking cotrimoxazole up to 12 weeks from ART initiation with 46% (14%–63%) reduction sustained 12–72 weeks
Possible benefit of azithromycin			
REALITY	Hakim (2017) [10,13]	1,805 ART-naïve participants with HIV ≥5 years old, with CD4 < 100 cells/μL starting ART randomized to enhanced prophylaxis (12 weeks IPT, 12 weeks fluconazole, 5 days azithromycin, single-dose albendazole, and continuous cotrimoxazole) versus continuous cotrimoxazole alone; beyond 12 weeks, about half of patients in both arms were prescribed IPT	27% (95% CI 2%–45%) reduction in all-cause 24-week mortality in enhanced prophylaxis group compared to standard prophylaxis group; greater risk of death from unknown cause in standard prophylaxis group (6% versus 3.8%, *p* = 0.03) and azithromycin may have prevented deaths due to serious bacterial infections (baseline CrAg-positive status was rare in unknown deaths, suggesting most of these deaths were unlikely to be due to cryptococcal disease)
Benefit of CrAg screening			
REMSTART	Mfinanga (2015) [14]	1,001 ART-naïve adults with HIV and CD4 <200 cells/μL in 6 clinics in Tanzania and Zambia randomized to ART initiation with CrAg screening and targeted preemptive fluconazole (if lumbar puncture CrAg testing negative or refused lumbar puncture) and community-based ART support or standard clinic-based care alone with ART initiation	28% (95% CI 10%–43%) lower mortality in the CrAg screening and community-based ART support group compared to the standard care group
Benefit of LAM screening **			
—	Lawn (2012) [15]	Cross-sectional study of ambulatory adults with HIV in South Africa evaluated for TB with sputum microscopy, culture, Xpert MTB/RIF, and urine LAM (complete test results in 516 patients)	Compared to reference of sputum culture, among patients with CD4 <100 cells/μL urine LAM was 51.7% (95% CI 32.5%–70.6%) sensitive in diagnosing TB, combination LAM and sputum microscopy 65.5% (95% CI 45.7%–82.1%) sensitive, and combination LAM and sputum Xpert MTB/RIF 75.9% (95% CI 56.5%–89.7%) sensitive; high specificity
**—**	Nakiyingi (2014) [16]	Prospective diagnostic accuracy study of 1,013 adults from Uganda with HIV and at least one symptom of TB (fever, cough, night sweats, weight loss) evaluated for TB with sputum microscopy, culture, mycobacterial blood cultures, and urine LAM	Compared to reference of culture-positive TB, among patients with CD4 <100 cells/μL urine LAM was 59.2% (95% CI 52.0%–66.1%) sensitive
—	Peter (2016) [17]	2,569 hospitalized adults in South Africa, Tanzania, Zambia, and Zimbabwe with HIV and at least one symptom of TB (fever, cough, night sweats, self-reported weight loss) randomized to available sputum-based TB testing (smear microscopy, culture, Xpert MTB/RIF) or sputum-based testing plus urine LAM	18% (95% CI 4%–30%) reduction in all-cause mortality up to 8 weeks in the LAM group compared to the no LAM group; percentage of participants starting ATT higher and time-to-initiation of ATT shorter in LAM group compared to no LAM group
STAMP	Gupta-Wright (2018) [18]	2,600 hospitalized adults with HIV in South Africa and Malawi randomized to TB screening regardless of symptoms with sputum-based Xpert MTB/RIF, urine-based LAM, and urine Xpert MTB/RIF or sputum-based Xpert MTB/RIF alone	Non-significant reduction in all-cause mortality up to 8 weeks in urine and sputum-based screening group compared to sputum screening alone; in participants with CD4 <100 cells/μL, 7.1% (95% CI 0.4%–13.7%) absolute difference in mortality in urine plus sputum-based screening group; almost half of patients could not expectorate sputum and urine LAM significantly improved TB diagnosis
Benefit of enhanced prophylaxis package where CD4 testing is available but CrAg and LAM screening unavailable			
REALITY	Hakim (2017) [10]	1,805 ART-naïve participants with HIV ≥5 years old, with CD4 < 100 cells/μL starting ART randomized to enhanced prophylaxis (12 weeks IPT, 12 weeks fluconazole, 5 days azithromycin, single-dose albendazole, and continuous cotrimoxazole) versus continuous cotrimoxazole alone; beyond 12 weeks, about half of patients in both arms were prescribed IPT	27% (95% CI 2%–45%) reduction in all-cause 24-week mortality in enhanced prophylaxis group; 33% (95% CI 7%–51%) reduction in incident TB up to 48 weeks; 62% (95% CI 17%–82%) reduction in incident cryptococcal meningitis up to 48 weeks
No benefit of empiric ATT			
REMEMBER	Hosseinipour (2016) [9]	850 ART-naïve participants with HIV ≥13 years old, with CD4 < 50 cells/μL, and without clinical evidence of TB (suspected or confirmed) from 10 high-TB-burden countries randomized to empiric ATT and ART or IPT and ART	Empiric ATT did not reduce 24-week all-cause mortality
STATIS	Blanc (2018) [19]	1,047 ART-naïve adults with HIV in Cambodia, Ivory Coast, Uganda, and Vietnam with CD4 < 100 cells/μL randomized to empiric ATT or baseline TB screening (sputum Xpert MTB/RIF, urine LAM, and CXR) and symptoms-based repeat TB screening during follow-up	Empiric ATT did not reduce 24-week all-cause mortality or incidence of invasive bacterial disease; empiric ATT associated with higher risk of grade 3–4 drug-related toxicity

* A Cochrane systematic review on cotrimoxazole prophylaxis showed decreased risk of death and serious bacterial infections in patients with both early and advanced HIV [20].

** A Cochrane systematic review on diagnostic accuracy of LAM also reported pooled diagnostic accuracy from published studies [21].

Abbreviations: ART, antiretroviral therapy; ATT, anti-TB therapy; CDC, Centers for Disease Control and Prevention; CI, confidence interval; CrAg, cryptococcal antigen; CXR, chest X-ray; IPT, isoniazid preventive therapy; LAM, lipoarabinomannan; TB, tuberculosis; WHO, World Health Organization.

## Current strategies are inadequate for identifying and preventing OIs and related deaths in late presenters

In the “treat all” era, the challenge is targeting effective prophylaxis against the bacterial, mycobacterial, and fungal pathogens that are the major causes of death in these late presenters. Such prophylaxis saves lives (see below) but also costs money and raises the potential risk of antimicrobial resistance if used indiscriminately. Further, when prophylaxis is provided conditional on a diagnostic test result, unless truly point-of-care, there is the potential for delays in ART initiation, which may substantially increase mortality risk.

Specific tests that have evidence supporting their use in reducing mortality and are recommended to target treatment/prophylaxis to patients with HIV and low CD4 cell counts include tuberculosis (TB) smear, Xpert MTB/RIF and urinary lipoarabinomannan (LAM) for TB, and cryptococcal antigen (CrAg) to detect cryptococcal infection (Table 1). Theoretically, all can be point-of-care tests; practically, however, some (particularly CrAg) are usually performed in a laboratory, and many or most are unavailable at remote health centers.

CD4 has consistently been found to be a key predictor of mortality after ART initiation [22], stronger than pre-ART viral load where both are available. Most HIV high-burden countries recommend baseline CD4 testing in national guidelines, although a few explicitly state that baseline CD4 testing is not a requirement for starting ART, as do WHO guidelines for rapid ART initiation [6,23–26]. To expedite ART, the donor program interpretation of WHO guidelines around CD4 cell count testing prior to the initiation of treatment has been to reduce funding for CD4 testing to shift funds toward viral load testing [27]. Many President’s Emergency Fund for AIDS Relief (PEPFAR)-funded programs are no longer recommending CD4 testing and/or do not pay for these tests, meaning that point-of-care machines are lying idle, and the proportion of patients receiving a CD4 count at presentation, providing results on which clinical decision-making can occur, is decreasing. For example, the International epidemiology Databases to Evaluate AIDS (IeDEA) Cohort Consortium has found a downward trend in baseline CD4 T-cell count testing (defined as testing within 3 months of enrollment and no later than 1 week after ART initiation) among patients in the East and southern Africa regions [28]. Similarly, after adopting a test-and-treat strategy in 2016, the number of reported CD4 tests in Malawi declined over 80% within one quarter of a year, from 15,207 tests during the quarter prior to test and treat to 2,548 tests during the quarter after adoption [29]. Many clinical criteria for identifying the most common treatable OIs (e.g., TB, cryptococcal meningitis), such as 2 weeks of cough, have low positive predictive value, meaning that they identify large numbers without these conditions. Further, clinical criteria to predict low CD4 and/or those at highest mortality risk are neither specific nor sensitive [5]. In the REALITY trial, despite a median CD4 of only 37 cells/μL and all participants having advanced HIV with CD4 < 100 cells/μL, only 53% of patients had WHO stage 3 or 4 disease; the other half had few or no discernible clinical symptoms or signs of advanced HIV disease and could not have been identified using clinical criteria alone [10].

## A practical resource-based approach for targeting OIs in the “treat all” era

**Fig 1:**
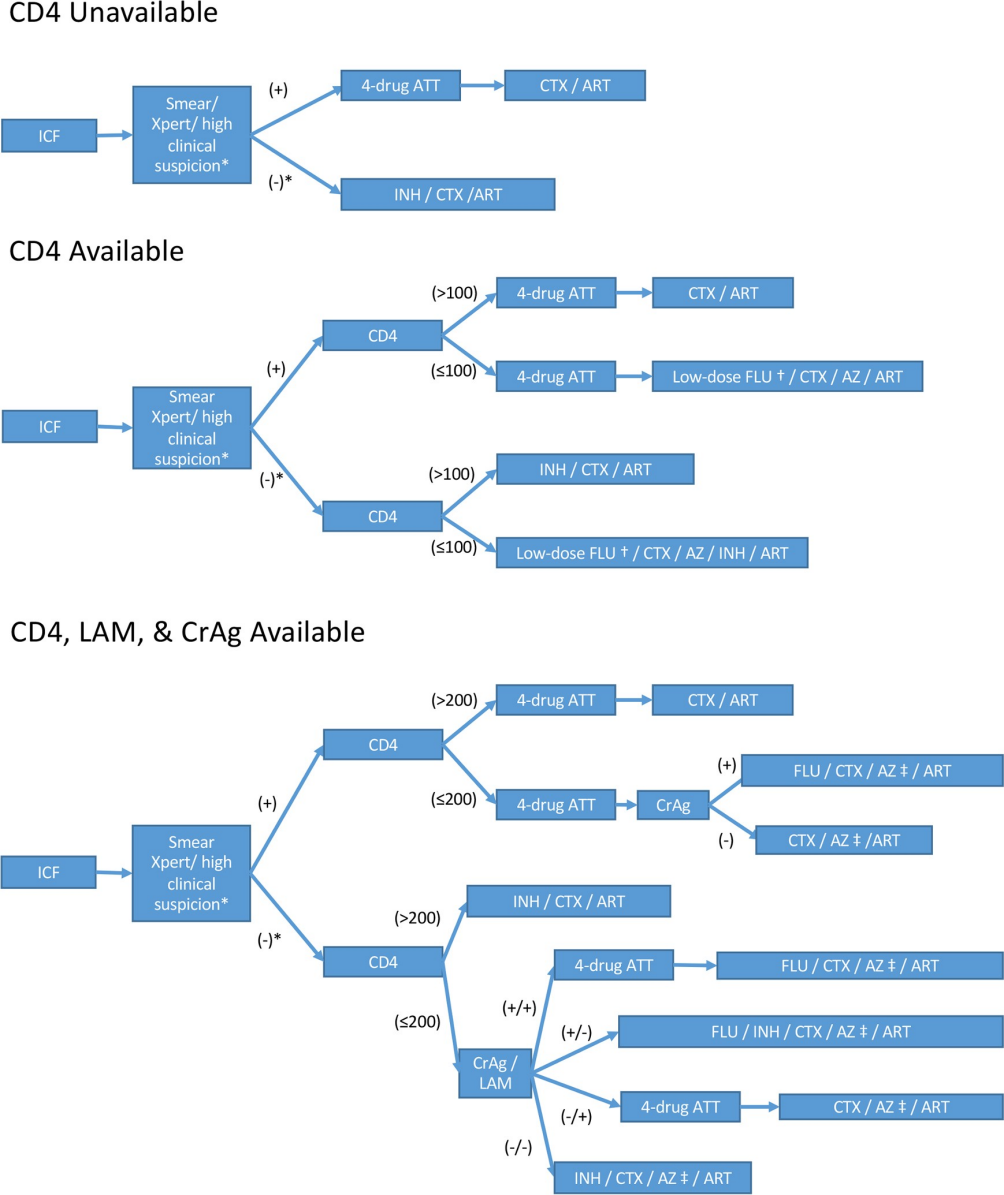
Resource-based approach for targeting OIs. * If smear or Xpert MTB/RIF is negative or unable to perform but suspicion for TB is high, consider further evaluation and/or empiric 4-drug treatment. † Low-dose FLU 100 mg/day for 12 weeks used in REALITY trial, compared to FLU 800 mg/day for 10 weeks then 200 mg/day maintenance pending CD4 count recovery recommended by WHO for CrAg-positive adults. ‡ AZ if CD4 ≤ 100 following REALITY trial. ART, antiretroviral therapy; ATT, anti-TB therapy; AZ, azithromycin; CrAg, cryptococcal antigen; CTX, cotrimoxazole; FLU, fluconazole; ICF, intensified TB case finding; INH, isoniazid; LAM, lipoarabinomannan; OI, opportunistic infection; TB, tuberculosis; WHO, World Health Organization.

Given the limited ability of clinical assessment alone to identify patients with advanced HIV disease and the evidence for mortality benefit of enhanced OI screening in patients with advanced disease (Table 1), we suggest that guidelines should take the availability of various point-of-need tests at sites into consideration in a resource-based approach (Fig 1).

### Scenario 1: Preventing OIs in settings where rapid CD4 testing is not feasible

CD4 testing remains important for staging HIV pre-ART, as it is the only way to identify the population at highest risk of death. However, even where immediate CD4 testing is unavailable, particularly in sub-Saharan Africa (SSA), all algorithms should still begin with intensive case finding for TB. Same-day screening with either smear or Xpert MTB/RIF should ideally be performed, but absence of such testing should not be a barrier to prompt ART initiation with isoniazid and cotrimoxazole (preferably as a fixed-dose-combination with pyridoxine [30]) when clinical suspicion for active TB disease is not high.

The TEMPRANO trial, which had a predominance of patients with early HIV (CD4 > 350 cells/μL), demonstrated the added benefit of 6 months’ isoniazid prophylaxis to ART (35% reduction in death or severe HIV-related illness), as did the REMEMBER trial in participants with advanced HIV disease (CD4 < 50 cells/μL); in both trials, mortality rates in the isoniazid treatment arm were lower than historical controls [2,9]. Of note, empiric 4-drug anti-TB treatment in those without pulmonary symptoms was neither superior to isoniazid prophylaxis alone [9] nor better than repeated screening if symptoms develop [19]. In an outpatient setting in 4 countries in SSA, the TB Fast Track Trial did not show a mortality benefit from empiric 4-drug anti-TB regimens in patients identified as being at high TB risk in the absence of CD4 testing [31]. The PROMPT study showed autopsy evidence of disseminated TB even among patients empirically treated with 4-drug anti-TB regimens prior to ART initiation and provides further evidence that empiric anti-TB therapy in the severely immunosuppressed does not improve outcomes [32]. In addition to isoniazid, across CD4 strata cotrimoxazole prophylaxis also reduces mortality and risk of serious bacterial infections and malaria within endemic regions [11,12] and is recommended regardless of CD4 count in areas with a high prevalence of severe bacterial infections and/or malaria [33].

### Scenario 2: Rapid CD4 count available without additional OI screening

Several assays (including Alere’s PIMA and Becton Dickinson’s FACSPresto) are commercially available for rapid CD4 testing. If rapid CD4 testing is available and results are <100 cells/μL, where other tests are not available, low-dose (100 mg/kg) fluconazole and a 5-day course of azithromycin (500 mg/day) along with isoniazid/cotrimoxazole/pyridoxine (as a late-presenters “package”) decreased mortality risk by 27% (3% absolute risk reduction) in the REALITY trial [10].

### Scenario 3: Rapid CD4 count testing available along with LAM and CrAg

Where rapid CD4 testing is available and additional tests for OIs can be performed, the challenge is balancing the excess mortality risks associated with potential delays to ART while waiting for test results against benefits of targeting prophylaxis more narrowly. In hospitalized patients with advanced HIV, urinary LAM testing followed by 4-drug anti-TB treatment for positives decreased 6-month mortality [18,17]. LAM is easy to obtain and can diagnose TB within minutes; sensitivity is highest in patients with low CD4 counts, who are more likely to have disseminated disease and are also often unable to produce adequate sputum samples for TB testing [18,16,21,15]. Next-generation assays with improved sensitivity are currently being evaluated (such as assays from Fujifilm and the Foundation for Innovative New Diagnostics [FIND] and from Salus Discovery) and may improve the future diagnostic yield of urinary LAM.

Immunosuppressed patients also benefit from CrAg screening and preemptive therapy with fluconazole if positive, when bundled with ART initiation. The REMSTART trial, which included participants with CD4 < 200 cells/μL, found that CrAg screening coupled with community-based ART support decreased mortality by 28% (4% absolute reduction) [14]. The IMMY CrAg lateral flow assay is highly sensitive, cheap, rapid, and validated on fingerstick capillary blood for point-of-care testing [34]. Early asymptomatic cryptococcal meningitis is common, and ideally, patients who test positive for CrAg should be offered a lumbar puncture to rule out meningitis and need for amphotericin-based therapy [35]. Those not diagnosed with meningitis should be started on high-dose fluconazole with ART deferred for 2 weeks to reduce the risk of life-threatening immune reconstitution inflammatory syndrome [36,37]. Overall, the proportion of CrAg-positive patients is relatively low even in those with low CD4 counts (estimated pooled prevalence 6.5% [95% CI 5.7%–7.3%] if CD4 < 100 cells/μL in 54 studies) [38], so expeditious ART initiation should be prioritized in those with low CD4 counts if CrAg testing is likely to result in significant delays in initiation, e.g., in many laboratory-based CrAg screening programs [39]. Alternatively, immediate low-dose fluconazole prophylaxis could be initiated, as in centers without tests available, and stopped if needed when results are available.

Based on the REALITY trial [10], 5 days of azithromycin can also be considered for patients with low CD4 counts in settings where additional tests for OIs are available. Deaths of unknown causes mostly occurred very soon after starting ART and were significantly lower (3.8% versus 6.0%, *p* = 0.03) in patients receiving enhanced prophylaxis including azithromycin; this difference may have been due in part to reductions in bacterial sepsis. Among those dying from unknown causes, baseline CrAg prevalence was very low, suggesting that these were not due to undiagnosed cryptococcosis [13].

## Implementation challenges and emerging diagnostics

Baseline CD4 testing is still important to guide the diagnostic evaluation for common OIs and prophylaxis in patients newly diagnosed with HIV. Conventional laboratory-based CD4 testing using flow cytometry requires substantial infrastructure and technical expertise and may lead to significant delays in obtaining actionable results, with a systematic review estimating a mean time of 10.5 days from the time a CD4 test was conducted to the time the result was received, versus 0.1 days with point-of-care CD4 testing [40]. Several point-of-care CD4 assays are available but require specialized instruments [41]. Alere PIMA has been extensively validated and is most commonly used for rapid CD4 testing [42]. Assays are needed that, like the IMMY CrAg lateral flow assay, meet WHO ASSURED criteria (affordable, sensitive, specific, user-friendly, rapid and robust, equipment-free, and deliverable to end users). Several emerging CD4 assays are in development or undergoing validation [43]. Omega Diagnostics’ VISITECT CD4 Advanced Disease assay is an instrument-free point-of-care lateral flow assay that provides a semiquantitative result for a CD4 count above or below 200 cells/μL and is currently being evaluated in SSA and India. This test, if accurate, could potentially be combined with point-of-care CrAg testing of venous or capillary whole-blood and urinary LAM testing for those with low CD4 counts even in remote health centers. The United Nations Children’s Fund (UNICEF) and Rhodes University (Grahamstown, South Africa) have also collaborated on a colorimetric aptamer-based CD4 reader that uses a personal cell phone device and mobile application to deliver CD4 test results.

## Conclusion

Baseline CD4 is an essential part of HIV care, and implementation research is needed to better streamline this and other new point-of-care tests for OIs to make them practical to perform in underresourced centers. We present a resource-based public health approach according to diagnostic test availability that could decrease early mortality after ART initiation and would be practical to implement. Our approach does not allow “the best to be the enemy of the good.” Even the most resource-constrained settings can immediately implement interventions that have the potential to save thousands of lives, and further refinement can be offered in settings where rapid screening for common OIs is feasible. An optimal approach requires that pre-ART CD4 (preferably as a simple point-of-care threshold test) continues to be available. We believe this provides a pragmatic algorithm to avoid delaying ART for the most immunosuppressed patients who are at the highest risk of dying.

## Abbreviations

ART: antiretroviral therapy
CDC: Centers for Disease Control and Prevention
CrAg: cryptococcal antigen
CXR: chest X-ray
FIND: Foundation for Innovative New Diagnostics
ICF: intensified TB case finding
IeDEA: International epidemiology Databases to Evaluate AIDS
IPT: isoniazid preventive therapy
LAM: lipoarabinomannan
LMIC: low- and middle-income country
OI: opportunistic infection
PEPFAR: President’s Emergency Fund for AIDS Relief
SSA: sub-Saharan Africa
TB: tuberculosis
UNICEF: The United Nations Children’s Fund
WHO: World Health Organization
